# Reply to Christelli *et al.*: Implementation of new Westgard Rules suggested by the Westgard Advisor software for five immunological parameters. What Six Sigma, quality control, the analytical Sigma-metric, and Westgard Advisor can and cannot do

**DOI:** 10.11613/BM.2025.020401

**Published:** 2025-06-15

**Authors:** Sten Westgard, Hassan Bayat

**Affiliations:** 1 Westgard QC Inc., Madison, USA; 2 Immunogenetics Research Center, Sina Medical Laboratory, Qaem Shar, Iran

**Keywords:** analytical Sigma-metrics, quality control, Six Sigma, Westgard Rules

## Abstract

The study of Cristelli *et al.* attempted to find fault with the rules suggested by Westgard Advisor software, claiming that implementing those rules did not improve the method performance. A fundamental misunderstanding of the utility and purpose of the analytical Sigma-metric and QC rules needs to be clarified.

## Graphical abstract

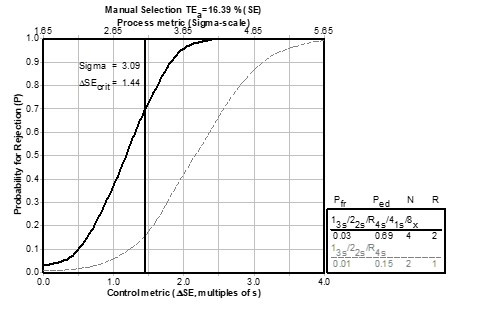

## Highlights

• Cristelli *et al*. implemented quality control (QC) rules suggested by Westgard Advisor, but found that implementation of these rules did not improve method performance

• QC rules and analytical Sigma metrics, by themselves, cannot improve method performance

• The function of the analytical Sigma-metric is to indicate performance of a method in a stable state and the function of QC is to detect errors

• The rules suggested by Westgard Advisor increased the error detection over the previous rules, accomplishing the purpose of Westgard Advisor and QC in general

## To the editor,

We read the study by Cristelli *et al*. with great interest and we would like to offer clarification about the purpose of the Westgard Advisor, quality control (QC) rules in general, as well as the analytical Sigma-metric itself (*1*).

First, let us disclose that we do not receive revenue from the Westgard Advisor software, which is wholly owned by Bio-Rad (Bio-Rad Laboratories, Hercules, USA). More than 15 years ago, Westgard QC Inc., sold the software algorithms that provide the basis to Westgard Advisor to Bio-Rad. Those algorithms, since, became available to the public domain.

The analytical Sigma-metric is an indicator of the stable state (in-control) performance, and QC is the detector of unstable (out-of-control) performance, rather than tools that directly improve a process. There appears to be some confusion over what a statistic is able to do:

A statistic, on its own, cannot improve (or degrade) a method’s stable performance.

An analytical Sigma metric, on its own, cannot improve the performance of a method.

Quality control, on its own, cannot improve the performance of a method.

If that’s hard to understand, think of it this way:

A smoke detector, and an alarm coming from the detector, on their own, cannot make a house less flammable or put out the flames.
A speedometer, and the reading of excessive speed, cannot make a car drive slower or more safely.
A hemoglobin A1c test and its test result, cannot cure diabetes.

All of these things are indicators of stable state and detectors of unstable state - that is, they can indicate how well a system performs in-control, and can detect when there is a problem and the system is out-of-control, allowing the operator to intervene. However, they must be acted upon, they cannot act by themselves. If a better performance in the stable state is desired, method improvement activities must be applied; and if a better detection of unstable state is desired, then QC performance must be improved. The intervention to improve analytical performance should be monitored by increase in Sigma-metric; and the intervention of improving QC should be monitored by the increase in probability of error detection (P_ed_). Cristelli *et al.* appear to expect that simply by changing the internal quality control (IQC) procedure, the method will improve.

When facing poor performance, like *“the ones with a lower analytical performance than the other parameters measured with the nephelometer”* in the study, the first step is to try improving the performance *via* reducing bias and/or imprecision. If it is not possible to improve performance, then QC must be increased. There is a trade-off between the quality of the stable state of the performance and the amount of QC needed. With a high-quality method, there is more space for a shift in performance (*i.e.*, the Critical-Error is higher) and therefore a less-demanding QC procedure is needed. In contrast, with a lower performing method, the Critical-Error is small and a more robust QC procedure is needed (*2*). The suggestions of the Westgard Advisor, and more fundamentally, the suggestions of an analytical Sigma-metric, can point to the need for an increase in QC rules. The impact of more QC rules is not an improvement in method performance, but an improvement in the error detection of the QC. That is, the analytical Sigma-metric can identify poor performance, which means more errors may be occurring, which then requires more vigilant QC efforts.

A more appropriate way to assess the impact of Westgard Advisor is to assess the changes in error detection caused by the change in the recommended QC rules, which can be assessed with Critical-Error graphs. More error detection means that the aim of the Cristelli *et al.* study is met, and the new QC rules *“are more efficient in the monitoring of analytical performance than those previously in use.”* In turn, this leads to a more effective prevention of the release of bad results. This improves patient safety *via* appropriate QC design.

**Figure 1:**
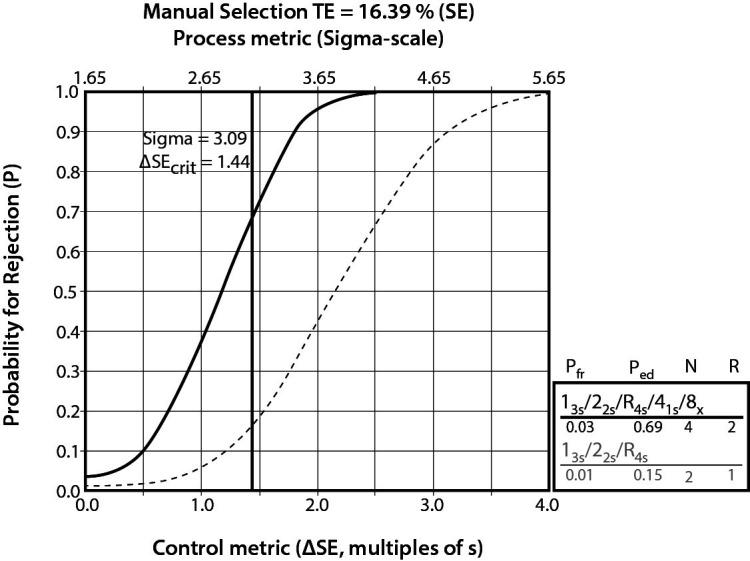
A Critical-Error graph showing the difference in error detection between Phase A and Phase B QC rules.

Let’s take the example of alpha-1 antitrypsin (AAT) level 3, where the Westgard Advisor recommended increasing the previous multirule from 1:3s/2:2s/R:4s (Phase A) to a full set of Westgard Rules 1:3s/2:2s/R:4s/4:1s/10:x (Phase B) (Figure 1).

Notice that the beginning procedure had an error detection of 15% (see the column under P_ed_ in the key), while the recommendation increases that P_ed_ to 69%. Thus, the new recommendation quadruples the ability to catch significant errors. There is a tradeoff, however, the P_fr_ increases from 1% to 3%.

A further confusion occurs in the study, due to the findings nevertheless presenting a rejection of the authors’ intended outcome.

The authors state, “*[T]here were inhomogeneous improvements in the three statistical values.*” In other words, three of the analytes in Phase D showed improvement in analytical Sigma metrics over Phase A, while two of the analytes declined. By most standards, when 3 of 5 analytical Sigma metrics increase, it is generally considered a successful improvement in performance. The authors attribute this improvement to improved staff handling of QC than of actual method improvement: “*Because of the study, the working behavior of the laboratory technicians changed noticeably for the better. Much more attention was paid to the correct handling of the IQC.*”

We are inclined to agree with the authors, that the improvements in Sigma metrics over the study period were due to issues other than actual analytical performance improvement, akin to the famous Hawthorne effect, since the Sigma metric itself cannot improve a method. We congratulate the laboratory on their now-better-trained staff.

To conclude, the analytical Sigma-metric can indicate improvements in analytical performance at stable state from recalibration to mitigate bias, the removal of sources of variation to mitigate imprecision. Distinctly, a Critical-Error graph can demonstrate the change in QC performance caused by a change in QC rules. The Cristelli *et al.* study correctly applied an intervention to improve the QC performance with more demanding QC rules, in order to better monitor lower analytical performance. However, it was incorrect to expect a detector to improve method performance.

## Data Availability

Data is available upon request.
